# Is Sunshine Vitamin Related to Adolescent Depression? A Cross-Sectional Study of Vitamin D Status and Depression Among Rural Adolescents

**DOI:** 10.7759/cureus.34639

**Published:** 2023-02-05

**Authors:** Pradeep Tarikere Satyanarayana, Ravishankar Suryanarayana, Susanna Theophilus Yesupatham, Sudha Reddy, Navya Reddy

**Affiliations:** 1 Community Medicine, Sri Devaraj Urs Medical College (SDUMC) Sri Devaraj Urs Academy of Higher Education and Research (SDUAHER), Kolar, IND; 2 Biostatistics, Sri Devaraj Urs Medical College (SDUMC) Sri Devaraj Urs Academy of Higher Education and Research (SDUAHER), Kolar, IND; 3 Biochemistry, Sri Devaraj Urs Medical College (SDUMC) Sri Devaraj Urs Academy of Higher Education and Research (SDUAHER), Kolar, IND; 4 Pediatrics, Sri Devaraj Urs Medical College (SDUMC) Sri Devaraj Urs Academy of Higher Education and Research (SDUAHER), Kolar, IND; 5 Psychiatry, Sri Devaraj Urs Medical College (SDUMC) Sri Devaraj Urs Academy of Higher Education and Research (SDUAHER), Kolar, IND

**Keywords:** rural teenagers, bdi-ii, vitamin d deficiency, adolescents, depression

## Abstract

Background: Adolescence is the phase of rapid transition of the body. The requirement of all minerals and vitamins changes in this phase of life so does Vitamin D. Despite Vitamin D being abundantly available, its deficiency, which can cause innumerable side effects on the body, is extremely common among the general population.

Material and methods: The present study was a cross-sectional study carried out from January 2021 to July 2022 for two years at various government rural high schools in Kolar, Karnataka, India. All adolescents who were aged 11-18 years and studying in 9^th^ and 10^th^ standards were included in the study after consent and assent. Adolescent boys and girls with any pre-existing mental health illness were excluded from the study. To assess depression, Beck's Depression Inventory (BDI-II) was used. Vitamin D3 levels were assessed by using VITROS Immunodiagnostic products using a 25-OH Total reagent pack. All data were entered in a Microsoft Excel sheet (Redmond, USA) and analyzed using IBM Corp. Released 2013. IBM SPSS Statistics for Windows, Version 22.0. Armonk, NY: IBM Corp. To check for the association between factors, Chi-square was applied with a level of significance defined as a p-value less than 0.05.

Results: Out of 451 students, 272 (60.3%) belonged to the 15-year age group, 224 (49.7%) were boys, 235 (52.1%) were studying in 10^th^ standard, 323 (71.6 %) belonged to nuclear families, 379 (84%) were non-vegetarian by diet, 222 (49.2%) had sun exposure in the afternoon, and 156 (34.6%) had a sun exposure of fewer than 60 minutes, 133 (29.5%) had severe depression according to Beck's Depression Inventory-II. One hundred sixty-two (35.9%) had insufficient Vitamin D3 levels (12-20 ng/ml), and 66 (14.6%) had deficient levels of Vitamin D3 (less than 12 ng/dl). There was a statistically significant association between depression and Vitamin D3 levels.

Conclusion: There are innumerable causes of adolescent depression. The present study shows Vitamin D levels were statistically associated with depression among adolescents. Vitamin D supplementation of at least 600 international units, which is the recommended dietary allowance (RDA), could be beneficial in tackling Vitamin D to sufficiency status (20-100 ng/ml) and also indirectly address Adolescent Depression. Better study designs, like randomized control trials showing Vitamin D intervention and its possible curative role in adolescent depression, are required to establish the causal association.

## Introduction

Traditionally labeled as sunshine Vitamin, Vitamin D is endogenously produced in the skin when exposed to Ultraviolet B rays. Few food products like dairy products, eggs, fish, and cod liver oil also contain natural vitamin D. In most countries, exiguous foods like milk and cheese are also fortified with Vitamin D [1]. Despite this abundantly available Vitamin D, its deficiency is extremely common among the general population. A few reasons identified for this possible silent epidemic in various parts of the world and also in a tropical country like India where there is abundant sunshine were body covering habits due to religious beliefs, staying indoors for the majority of daytime with little or no physical activity, lack of open spaces and direct access to sunlight in high human density habitations resulting in the high prevalence [2,3]. Of various above-mentioned factors, a few pieces of research also mention obesity as a possible risk factor for Vitamin D deficiency among adolescents and young individuals. The proposed hypothesis for Vitamin D deficiency is more absorption of fat-soluble Vitamin D into adipose tissue [4]. Added to this, seasonal Vitamin D deficiency also has an inconsequential role [5].

Adolescence is the phase of rapid transition of the body. The requirement of all minerals and vitamins changes in this phase of life so does Vitamin D. The health implications of Vitamin D in terms of bone health are increasingly understood, yet its impact, particularly on mental health, is unclear. Although recent data has shown rangy corroboration that Vitamin D has an important impact on the pathophysiology and progression of serious chronic illness, especially on mental health. Contemporary evidence has been established that stunted Vitamin D levels are associated with depression, poor mood, and other mental disorders [6]. Individuals with normal levels of Vitamin D, which is 30-100 ng/dl, have a much lower probability of developing depression [7-9]. A study done in Norway has shown that Vitamin D deficiency is very common among psychogeriatric patients, independent of the diagnostic category [10]. A study done on the elderly showed that Low 25(OH)D was independently associated with a greater increase in depressive symptom scores and incident depression in community-dwelling older adults [11]. With this background, the study was started to find out the association between depression and Vitamin D status among rural adolescent boys and girls.

## Materials and methods

The present study was a cross-sectional study carried out from January 2021 to December 2022 for two years at various government rural high schools in Kolar, Karnataka, India. Twenty rural schools in Kolar were selected. A study done in India on school children has shown the prevalence of Vitamin D deficiency as high as 81%(p). With an error of 5% and 95% confidence interval sample size was calculated, which was 243 [12]. The sample size was calculated using Open Epi software Version 3.01. All adolescents who were aged 11-18 years studying in 9^th^ and 10^th^ standards were included in the study after consent and assent. Adolescent boys and girls with any pre-existing mental health illness, like already diagnosed severe depression or a history of any suicidal tendency or suicidal attempts in the past, were excluded from the study. To assess socio-demographic status, a pretested semi-structured questionnaire was used. To assess depression, Beck's Depression Inventory (BDI-II) was used, which is a 21 items Likert scale. According to Beck's Depression scale, the scores for each of the 21 questions are added up. The highest possible total for the whole test would be 63, and the lowest possible score for the test would be zero. Various categories of depression, according to Beck, would be based on summed-up scores, i.e., 0-10, which is considered normal, 11-16 is mild mood disturbance, 17-20 is borderline clinical depression, 21-30 is moderate depression, 31-40 is severe depression, and more than 40 is extreme depression [13]. The Indian Academy of Pediatricians (IAP) guidelines suggested cutoff for Vitamin D was used in the present study, which is less than 12 ng/dl as deficient, 12-20 ng/dl as insufficient, and more than 20 ng/ml as sufficient for a tropical country like India. The International Association of Endocrinology defined a vitamin D level of 21-29 ng/mL as insufficiency and less than 20 ng/ml as a deficiency [14]. All school children were interviewed by the Assistant professor from the department of community medicine, who had prior experience in using the BDI scale. Venous blood was taken by an experienced lab technician with all aseptic precautions, transported within the vaccine carrier box with the temperature well maintained according to the temperature range, and analyzed in Central Diagnostic Laboratory Services, Biochemistry Department, Sri Devaraj Urs Medical College, SDUAHER, Kolar. All precautions were taken to avoid any hemolysis of blood during fresh blood withdrawal and also during transportation. Vitamin D3 levels were assessed using VITROS immunodiagnostic products using a 25-OH total reagent pack. Students with clinical depression after the interview were referred to a psychiatrist for any further clinical intervention. The study was started after Central Ethics Committee approval (CEC SDUAHER/Res.Proj.173/2020-21). Informed written consent/assent was taken from the school children by informing them about the benefits and risks involved in the study. Autonomy was maintained for study participants making participation in the study voluntary. Confidentiality was also maintained as the participants’ names and personal details were not recorded.

## Results

Out of 451 adolescent rural school students, 272 (60.3%) were from the 15-year age group, 224 (49.7%) were boys, 235 (52.1%) were studying in 10^th^ standard, 323 (71.6 %) belonged to nuclear families (a family group consisting of parents and their children, typically living in one home residence) and rest belonged to joint families (a family that consists of two or more generations from the same paternal or maternal line that shares a home and lives together), 379 (84%) were non-vegetarian by diet whose diet contains meat which could be red, poultry, seafood, or the flesh of any other animal and rest of the school children were vegetarians who do not take any animal source of protein, 222 (49.2%) had sun exposure in the afternoon, and 156 (34.6%) had sun exposure of fewer than 60 minutes (Table 1).

**Table 1:** Distribution of adolescent school children according to various socio-demographic profile.

		Frequency	Percent
Age in years	14	50	11.1
	15	272	60.3
	16	117	25.9
	17	12	2.7
Gender	Boys	224	49.7
	Girls	227	50.3
Class studying	9	216	47.9
	10	235	52.1
Type of family	Nuclear	323	71.6
	Joint	128	28.4
Diet	Vegetarian	72	16.0
	Non-vegetarian	379	84.0
Timing of outdoor activity	Afternoon	222	49.2
	Evening	229	50.8
Duration	Less than 30 minutes per day	156	34.6
	More than 30 minutes per day	295	65.4

Out of 451 students, 90 (20%) had moderate depression, and 133 (29.5%) had severe depression, according to Beck's Depression Inventory-II (Table 2).

**Table 2:** Distribution of adolescent school children according to depression category as given by Beck's Depression Inventory–II

		Frequency	Percent
Beck's depression Inventory	Mild Mood disturbance	168	37.3
	Borderline Clinical depression	60	13.3
	Moderate	90	20.0
	Severe	133	29.5
	Total	451	100.0

Out of 451 students, 162 (35.9%) had insufficient Vitamin D3 levels, and 66 (14.6%) had deficient levels (Table 3).

**Table 3:** Distribution of adolescent school children according to Vitamin D levels.

		Cutoff values	Frequency	Percent
Vitamin-D3 Levels	Deficient	Less than 12 ng/ml	66	14.6
	Insufficient	12 to 20 ng/dl	162	35.9
	Sufficient	20-100ng/dl	223	49.4
	Total		451	100.0

63.1% of female adolescent school children had minimal depression, 71.1% of 10^th^ standard students had moderate depression, 63.7% of those adolescent school children who work out (exercise) during the evening had minimal depression, and all these factors had a statistically significant association with p-value less than 0.01 (Table 4). There was a statistically significant association between depression and Vitamin D3 levels (Table 5).

**Table 4:** Association between various factors and depression levels among adolescent school children.

BDI	Gender		Class		Family type		Diet		Timing	
	Male	Female	9	10	Nuclear	Joint	Vegetarian	Mixed	Afternoon	Evening
Mild Mood Disturbance	62(36.9%)	106(63.1%)	81(48.2%)	87(51.8%)	124(73.8%)	44(26.2%)	24(14.3%)	144(85.7%)	61(36.3%)	107(63.7%)
Borderline Clinical Depression	31(51.7%)	29(48.3%)	43(71.7%)	17(28.3%)	44(73.3%)	16(26.7%)	7(11.7%)	53(88.3%)	27(45.0%)	33(55.0%)
Moderate Depression	43(47.8%)	47(52.2%)	26(28.9%)	64(71.1%)	65(72.2%)	25(27.8%)	21(23.3%)	69(76.7%)	57(63.3%)	33(36.7%)
Severe Depression	88(66.2%)	45(33.8%)	66(49.6%)	67(50.4%)	90(67.7%)	43(32.3%)	20(15.0%)	113(85.0%)	77(57.9%)	56(42.1%)
P value*	0.001		0.001		0.64		0.17		0.001	

**Table 5:** Association between Vitamin D 3 levels and depression levels among adolescent school children.

		Vitamin D levels			P-value
		Deficient	Insufficient	Sufficient	
Becks Depression levels	Mild Mood Disturbance	39(23.2%)	49(29.2%)	80(47.6%)	<0.01
	Borderline Clinical Depression	6(10.0%)	14(23.3%)	40(66.7%)	
	Moderate Depression	10(11.1%)	35(38.9%)	45(50.0%)	
	Severe Depression	11(8.3%)	64(48.1%)	58(43.6%)	

Among adolescent school children, those who were studying in 9^th^ standard and exercising in the afternoon had higher odds of having mild depression. Among adolescent school children, those who were exercising in the afternoon had higher odds of having moderate depression. Among adolescent school children, those who were females and had Vitamin D deficiency had higher odds of having severe depression (Table 6).

**Table 6:** Multinomial logistic regression analysis of various factors with depression

BDI	Socio-demographic Factors	B	P value	Odds ratio	Lower Bound	Upper Bound
Borderline Clinical Depression	Male	.591	.085	1.807	.922	3.540
	9^th^ standard	1.427	.001	4.165	1.840	9.429
	Nuclear	.384	.309	1.468	.700	3.079
	Vegetarian	-.258	.582	.773	.309	1.932
	Afternoon	.967	.008	2.631	1.288	5.375
	Vitamin D Insufficiency	-.507	.341	.602	.212	1.711
	Vitamin D Deficiency	-.147	.711	.864	.397	1.877
Moderate Depression	Male	.152	.611	1.164	.648	2.090
	9^th^ standard	-.652	.071	.521	.257	1.057
	Nuclear	-.170	.598	.844	.449	1.585
	Vegetarian	.407	.249	1.503	.752	3.003
	Afternoon	.834	.009	2.302	1.234	4.296
	Vitamin D Insufficiency	-1.163	.010	.312	.129	.754
	Vitamin D Deficiency	-.051	.878	.950	.494	1.828
Severe Depression	Female	1.231	.001	3.423	2.002	5.853
	9^th^ standard	.738	.019	2.091	1.126	3.884
	Nuclear	-.189	.516	.828	.468	1.465
	Vegetarian	-.021	.951	.979	.495	1.936
	Afternoon	.994	.001	2.703	1.538	4.750
	Vitamin D Insufficiency	-.452	.293	.637	.274	1.478
	Vitamin D Deficiency	1.039	.001	2.826	1.562	5.115

## Discussion

The present study was a cross-sectional study carried out among rural adolescent school students for two years. Four hundred fifty-one rural high school students took part in the study. The majority were 15 years boys studying in 10^th^ standard. Students from nuclear families were common, 222 (49.2%) had sun exposure in the afternoon, and 156 (34.6%) had sun exposure for less than 60 minutes. Out of 451 rural high school students, 20% had moderate depression, and 29.5% had severe depression, according to Beck's Depression Inventory-II. The present study showed that Vitamin D deficiency had a statistically significant association with depression, with students who were studying in the 9^th^ standard and exercising in the afternoon having higher odds of minimal depression, those who were exercising in the afternoon having higher odds of moderate depression and those who were females and had Vitamin D deficiency (VDD) had higher odds of having severe depression. 

Studies have shown that Vitamin D-deficient people have increased odds of having clinically significant depression. Various studies conducted in different parts of the world suggest that irrespective of nutrition intake, longitudinal and latitudinal variation for sun exposure, skin pigmentation, and gender, there is a clear causal relationship between vitamin D status and depression among the healthy general population and establishing that Vitamin D is crucial to mental health [15-20]. Regardless of this sufficient evidence, biological mechanisms coupling Vitamin D levels and mental health status are still not fully understood. There is a shred of sizable evidence that neurons and glia in many parts of the brain, like the cingulate cortex and hippocampus have Vitamin D receptors which are involved in neuroimmunomodulation, regulation of neurotrophic factors, neuroprotection, neuroplasticity, and brain development, demonstrating that vitamin D might be associated with depression. The neoteric hypothesis proposes that an elevation in neuronal calcium level is a major component accountable for driving the onset of depression, where it is suggested that Vitamin D maintains calcium homeostasis and hence its deficiency may contribute to the onset of depression [21,22]. Oxidative stress and neuro-inflammation alterations cause invigoration of peripheral macrophages and central microglia, dysfunction of the hypothalamus-pituitary-adrenal (HPA) axis, and hypercortisolemia causing dendritic growth, synaptic plasticity, and deterioration in synaptic communication which is inhibited by abundant Vitamin D levels by secreting neurotransmitters, especially dopamine and exhibiting its neuro-modulatory and neuroprotective effects [23]. Cellular biology explains that the wide distribution of Vitamin D receptors and 1-α-hydroxylase throughout the brain allows for the local production of activated Vitamin D regulating the nerve growth factor and glial cell line-derived neurotrophic factor, which orchestrates the cellular architecture of the brain [24]. It is also said that activated Vitamin D has neuroprotective effects via neuromodulation, anti-inflammatory, anti-ischemic, and anti-oxidant properties. Other evidence is that Vitamin D induces the expression of the serotonin-synthesizing gene tryptophan hydroxylase 2 while repressing the expression of tryptophan hydroxylase 1, which plays a definite role in serotonin synthesis, establishing a thin link formation of serotonin and Vitamin D levels, thus fostering its supplementation might play a significant role in depression and its treatment [25,26]. Various systematic reviews and meta-analyses suggest that Vitamin D status is clinically and statistically associated with depression [27-29]. Most mental illnesses start at an early age, and the majority of cases are undiagnosed. The physiological impact of suboptimal nutrition on brain function is not fully understood, but adequate concentrations of both macro- and micronutrients are needed for optimal brain function. An evidence gap map has spotted the beneficial effect of Vitamin D on mental health conditions [30].

The strengths of the present study include a validated questionnaire that was used to assess depression among adolescent boys and girls. A standard diagnostic test was used to assess Vitamin D deficiency. The present study would be the first of this type to relate vitamin deficiency with depression, especially among rural adolescents. The study has many limitations. The study uses BDI, which assesses only symptoms of depression as a static measure. A relatively smaller sample size taken from the same geographic terrain hinders the generalization of study results. The temporal association between depression and Vitamin D deficiency (VDD) can only be established with better study designs which were not done in the present study as the present study was a cross-sectional study. There are various other factors like anemia, social factors like relation with father, mother, and school performance which could have played a role in depression, which were not assessed in the present study. The present study recommends Vitamin D supplementation at schools as the majority of adolescent students had VDD despite abundant sources.

## Conclusions

Vitamin D deficiency is extremely common among adolescents, and it remains unaddressed. Depression in adolescents is very common, and causes for depression in this age group could be many. With all evidence suggesting that Vitamin D is associated with depression, Vitamin D supplementation can be a metaphor for tackling adolescent depression. More evidence should be generated with a better study design to establish Vitamin D status and its role in depression, especially in adolescents.
